# Functional principal component analysis for identifying multivariate patterns and archetypes of growth, and their association with long-term cognitive development

**DOI:** 10.1371/journal.pone.0207073

**Published:** 2018-11-12

**Authors:** Kyunghee Han, Pantelis Z. Hadjipantelis, Jane-Ling Wang, Michael S. Kramer, Seungmi Yang, Richard M. Martin, Hans-Georg Müller

**Affiliations:** 1 Department of Statistics, University of California Davis, Davis, California, United States of America; 2 Departments of Pediatrics and of Epidemiology, Biostatistics and Occupational Health, McGill University, Montreal, Canada; 3 Bristol Medical School, Population Health Sciences, University of Bristol, Bristol, United Kingdom; 4 National Institute for Health Research Bristol Biomedical Research Centre, University Hospitals Bristol NHS Foundation Trust and the University of Bristol, Bristol, United Kingdom; Cincinnati Children’s Hospital Medical Center, UNITED STATES

## Abstract

For longitudinal studies with multivariate observations, we propose statistical methods to identify clusters of archetypal subjects by using techniques from functional data analysis and to relate longitudinal patterns to outcomes. We demonstrate how this approach can be applied to examine associations between multiple time-varying exposures and subsequent health outcomes, where the former are recorded sparsely and irregularly in time, with emphasis on the utility of multiple longitudinal observations in the framework of dimension reduction techniques. In applications to children’s growth data, we investigate archetypes of infant growth patterns and identify subgroups that are related to cognitive development in childhood. Specifically, “Stunting” and “Faltering” time-dynamic patterns of head circumference, body length and weight in the first 12 months are associated with lower levels of long-term cognitive development in comparison to “Generally Large” and “Catch-up” growth. Our findings provide evidence for the statistical association between multivariate growth patterns in infancy and long-term cognitive development.

## Introduction

### Objective of the study

The link between deficient growth in infancy and later life cognitive performance degradation has been widely accepted [1–3]. Stunting and faltering during infancy, or early childhood, are associated with reduced cognitive ability in later age performance [4, 5], and these growth patterns have been the subject of extensive investigation [6–9]. In most of the previous work, investigators have studied this association by examining single growth indicators, for example head circumference [10] or body weight [11]. In particular, [12] examined how cognitive development of children in Vietnam is associated with pre-defined growth features at age 1 year. While features such as stunting, underweight, wasting and small head circumference were examined, the previous analysis was based on one growth modality, for example body length or weight.

We propose a straightforward way to combine multiple growth indicators under a single framework. Our approach provides a comprehensive assessment of the potential risk in terms of cognitive development using longitudinal information from growth patterns of several growth modalities. Specifically, we demonstrate a data analysis procedure that combines measurements from three commonly recorded time-varying growth traits, head circumference, body length and body weight. We then identify growth patterns that can be associated with subsequently measured full-scale IQ (Wechsler abbreviated scale of intelligence, WASI). The simultaneous consideration of multiple trajectories is a main novel feature of our approach.

We also devise a simple method to learn archetypal growth patterns from data and examine their association with subsequent IQ outcomes. Our methods assess multiple growth indicators nonparametrically without the need of prior growth charts [13]. Using a functional data analysis (FDA) framework [14, 15], the proposed methodology combines multiple growth indicators and identifies data-driven clusters of infants according to their growth profiles. Recently, [16] and [17] considered quantile contour estimation of functional principal components (FPC) with emphasis on analysis for growth curves, but only with a single growth trajectory sample. In a related approach, [18] focused on finding subjective-specific warping functions to extract common features among multivariate growth traits. In contrast to existing approaches, we profile multiple growth patterns in terms of archetypal analysis [19–21], where we implicitly assume that extreme growth patterns can be used to represent individual growth curves in the sample through convex combination.

Our findings from applying the proposed methodology to the PROBIT growth study cohorts [22, 23] suggest that the proposed methodology is capable of identifying infant subgroups that differ in a statistically significant way in terms of the average level of associated IQs and thus can serve as a useful tool for identifying subgroups at risk of impaired cognitive development.

### Data description

**Fig 1:**
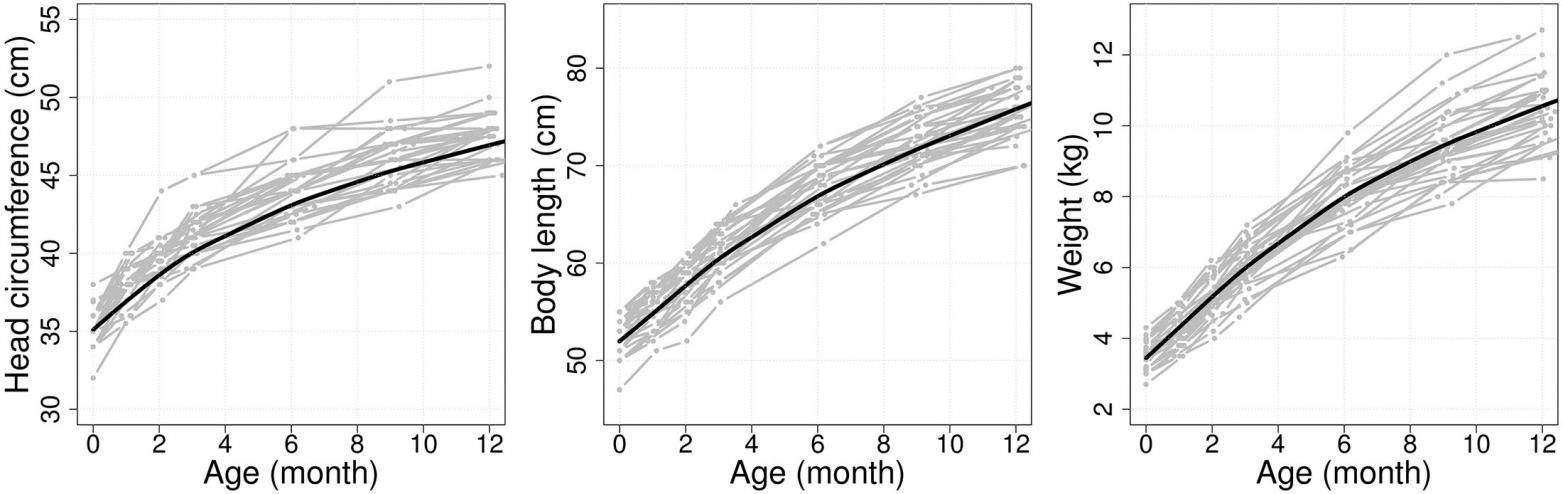
Irregular and sparse longitudinal observations from the PROBIT data. Head circumference (HC, left), body length (LN, middle) and weight (WT, right) are illustrated for a random selection of 30 subjects (gray) out of about 12,800 total children, along with estimated mean curves for each longitudinal trait (black solid lines).

The data were collected as part of WHO’s Promotion of Breastfeeding Intervention Trial (PROBIT) in the Republic of Belarus [22, 23]. They include growth measurements taken during the infancy of full term babies who weighed at least 2.5kg at birth among 17,045 total subjects. The physical traits recorded are head circumference, body length and body weight which were measured six times during the first year after birth. However, the sampling schedules were not strictly followed and some children did not have a full set of six measurements, the data are best characterized as irregularly sampled longitudinal observations and see Fig 1. For example, about 5.5% of the children have less than six measurements. For children with complete records, average measurement times were approximately 1.05 (0.12), 2.05 (0.13), 3.11 (0.24), 6.12 (0.31), 9.12 (0.34) and 12.10 (0.21) months after birth, where standard deviations at each visit are given in parentheses. We also refer to the design plot for measurement times during the first year in [13], which demonstrates the extent of sparsity and irregularity of the observation schedules over the first year after birth. The cognitive ability of children was assessed using the WASI score of the full-scale IQ measured at 6.5 years.

In addition to time-varying growth traits, a multitude of demographic covariates were also recorded. These covariates were used in later stages to control for potential confounding effects including socio-demographic factors. For example, children whose parents attended university have higher IQ measurements on average than children whose parents did not to complete high-school education. These covariates are known to affect later age IQ throughout a child’s infancy [24–27]. Other covariates included the sex of the child, maternal and paternal education levels, maternal and paternal age-at-birth, maternal smoking during pregnancy, duration of exclusive breast feeding and the hospital where the child was born. The sample size after preprocessing was *n* = 12,809 children, whose data were analyzed in our study. For details of data records and preprocessing, we refer to [13] and [22].

## Methodology

### Functional principal component analysis

Let *X*_*i*_(*t*) be a realization of a time-varying trait *X*(*t*) for the *i*-th subject, 1 ≤ *i* ≤ *n*, at each time point t∈T. Assuming independent measurements between subjects and that the *X*_*i*_(*t*) have smooth trajectories over time *t*, we apply functional principal component analysis (FPCA) to decompose patterns of temporal variation. A basic feature of FPCA is that the time-varying trait of the *i*-th subject admits the Karhunen-Loève expansion [28–30]

$$\begin{array}{c} X_{i}\left(t\right)=\mu \left(t\right)+\sum_{k\geq 1}\xi_{ik}\phi_{k}\left(t\right), \end{array}$$ (1)

where *μ*(*t*) = E*X*(*t*), and the *ξ*_*ik*_ are uncorrelated random variables with mean zero and variance λ_*k*_ satisfying λ_1_ ≥ λ_2_ ≥ ⋯. Here in Eq (1), the *ξ*_*ik*_ are the *k*-th FPC scores of the *i*-th subject, associated with the eigenfunction *ϕ*_*k*_ for all *k* ≥ 1. For theoretical background on FPCA and related techniques, see [15, 31–36].

In longitudinal studies, however, measurements of time-varying traits are only available at *N*_*i*_ successive time points, say $t_{i1}<\cdots <t_{iN_{i}}$, for the *i*-th subject. We note that the set of time points $\left\{t_{i1},\ldots ,t_{iN_{i}}\right\}$ may differ among the *n* subjects and *N*_*i*_ may be small. FPCA for longitudinal data has been widely investigated [14, 37–40]. Specifically, [41, 42] proposed a technique to perform FPCA for sparse longitudinal data, based on principal components analysis through a conditional expectation (PACE) scheme. Specifically, we consider sparse and noisy longitudinal observations ${\tilde{X}}_{ij}=X_{i}\left(t_{ij}\right)+\epsilon_{ij}$, instead of continuous and unperturbed observations of time-varying traits *X*_*i*_(*t*), where the *ϵ*_*ij*_ are independent mean zero measurement errors. By assuming that *ξ*_*ik*_ and *ϵ*_*ik*_ follow a joint normal distribution, the best linear predictors of the FPC scores *ξ*_*ik*_ are given by

$$\begin{array}{c} {\hat{\xi }}_{ik}={\hat{\lambda }}_{k}{\hat{\phi }}_{ik}^{⊤}{\hat{\Sigma }}_{{\tilde{X}}_{i}}^{-1}\left({\tilde{X}}_{i}-{\hat{\mu }}_{i}\right), \end{array}$$ (2)

where ${\tilde{X}}_{i}={\left({\tilde{X}}_{i1},\ldots ,{\tilde{X}}_{iN_{i}}\right)}^{⊤}$ are longitudinal observations, ${\hat{\mu }}_{i}={\left(\hat{\mu }\left(t_{i1}\right),\ldots ,\hat{\mu }\left(t_{iN_{i}}\right)\right)}^{⊤}$ are the estimates of mean vectors of $\text{E}{\tilde{X}}_{i}$, and ${\hat{\Sigma }}_{{\tilde{X}}_{i}}$ is the estimated *N*_*i*_ × *N*_*i*_ variance-covariance matrix of $\Sigma_{{\tilde{X}}_{i}}$ with (*j*, *ℓ*)-elements given by $\text{Cov}({\tilde{X}}_{ij},{\tilde{X}}_{i\ell })$. Also, $\left({\hat{\lambda }}_{k},{\hat{\phi }}_{k}\left(t\right)\right)$, *k* ≥ 1, are pairs of estimators for eigenvalues and eigenfunctions, which are the solutions of the following equations with respect to (λ_*k*_, *ϕ*_*k*_(*t*)),

$$\begin{array}{c} \begin{array}{cc} & \int_{T}G\left(s,t\right)\phi_{k}\left(s\right)\;ds=\lambda_{k}\phi_{k}\left(t\right)\;\left(k=1,2,\ldots \right),\\ & \;\;\text{subject}\;\text{to}\;\lambda_{k}\geq \lambda_{k+1}\;\text{and}\;\int_{T}\phi_{k}\left(t\right)\phi_{\ell }\left(t\right)\;dt=\{\begin{array}{cc} 0 & \left(k\neq \ell \right)\\ 1 & \left(k=\ell \right) \end{array}, \end{array} \end{array}$$ (3)

where *G*(*s*, *t*) = Cov(*X*(*s*), *X*(*t*)) is the auto-covariance function of *X*, so that we may write ${\hat{\phi }}_{ik}={\left({\hat{\phi }}_{k}\left(t_{i1}\right),\ldots ,{\hat{\phi }}_{k}\left(t_{iN_{i}}\right)\right)}^{⊤}$; see [43] and [15] for comprehensive overviews on FDA and recent developments in the interface between FPCA and longitudinal data.

Once we have estimated eigenfunctions ${\hat{\phi }}_{k}\left(t\right)$ through the PACE method in Eq (2), longitudinal patterns of ${\tilde{X}}_{i}$ can be summarized by the corresponding FPC scores ${\hat{\xi }}_{ik}$. In fact, unobserved time-varying traits *X*_*i*_(*t*) can be reconstructed as ${\hat{X}}_{i}\left(t\right)=\hat{\mu }\left(t\right)+\sum_{k=1}^{K}{\hat{\xi }}_{ik}{\hat{\phi }}_{k}\left(t\right)$, followed by the representation in Eqs (1) and (3) with a cut-off value *K* ≥ 1. The truncation point *K* can be chosen as the smallest value satisfying $\sum_{k=1}^{K}{\hat{\lambda }}_{k}/\sum_{\ell \geq 1}{\hat{\lambda }}_{\ell }\geq \kappa$ for a given 0 < *κ* < 1, so that a fraction *κ* of variance is explained (FVE), see [15, 30]. The infinite dimensional functions ${\hat{X}}_{i}$ will then be represented by *K*-vectors ${\left({\hat{\xi }}_{i1},\ldots ,{\hat{\xi }}_{iK}\right)}^{⊤}$, which provides the required dimension reduction.

### Identification of outlying subjects

To study archetypes in the multivariate data analysis framework, we cluster longitudinal observations ${\tilde{X}}_{i}$ into subgroups based on trajectory patterns of reconstructed time-varying traits *X*_*i*_(*t*). Time-varying traits are recovered by the first few FPC scores with high fraction of variance explained (FVE). In practice, the first two FPC scores produce relatively clear discrimination of the data characteristics in many sparse and irregular longitudinal studies [41, 42, 44, 45]. As an exploratory illustration tool for outlier detection in multivariate data analysis, the bagplot [46] was introduced as a generalization of the univariate boxplot. In the bagplot, halfspace location depth [47] is usually adopted so that the multivariate data points are ordered by an extended notion of univariate rank. The halfspace location depth $D(x;X_{n})$ of a point $x={\left(x_{1},\ldots ,x_{K}\right)}^{⊤}\in R^{K}$ over *K*-variate data $X_{n}=\left\{\xi_{i}\in R^{K}:1\leq i\leq n\right\}$ is defined by the smallest number of ***ξ***_*i*_ contained in any halfspace with boundary line passing **x**. Then, data points can be ordered by depth, that is $D\left(\xi_{i};X_{n}\right)\leq D\left(\xi_{i^{\prime }};X_{n}\right)$, 1 ≤ *i* ≠ *i*′ ≤ *n*. For modern concepts and related work on statistical depth, see also [48] and [49].

In this study, for the purpose of providing flexible inference based on sparse and irregularly observed functional and longitudinal data, we utilize the highest density region (HDR) as in [50] and [51]. We consider the (1 − *α*)-HDR for the *K*-variate data defined by

$$\begin{array}{c} R^{\xi }\left(1-\alpha \right)=\{x\in R^{K}:f_{\xi }\left(x\right)\geq f_{\alpha }\}\;\left(0<\alpha <1\right), \end{array}$$ (4)

where *f*_***ξ***_ is the joint density of a random vector ***ξ*** = (*ξ*_1_, …, *ξ*_*K*_)^⊤^ and

$$\begin{array}{c} f_{\alpha }=\arg\max\{y>0:\int_{R^{K}}f_{\xi }\left(x\right)\cdot I\left(f_{\xi }\left(x\right)\leq y\right)\;dx\leq \alpha \} \end{array}$$ (5)

in Eqs (4) and (5), respectively. Taking *α* = 0.05 yields a support region where observations are expected to fall with at least 95% probability. We also note that the HDR captures the nature of the distribution of the data like location, scale, correlation and tail information in a flexible manner. [52] proposed a kernel-type estimator of $R^{\xi }\left(1-\alpha \right)$, where *f*_***ξ***_ and *f*_*α*_ are replaced by kernel density estimators, respectively. For example, one can use ${\hat{f}}_{\xi }\left(x\right)=n^{-1}\sum_{i=1}^{n}\prod_{k=1}^{K}L_{h_{k}}\left(\xi_{ik}-x_{k}\right)$ with bandwidths *h*_*k*_ > 0, where *L*_*h*_(*v*) = *L*(*v*/*h*)/*h* is a scaled version of a baseline kernel *L* that is a probability density function with finite variance. The kernel estimator $\hat{R}\left(1-\alpha \right)$ of R(1-α) also enjoys level information of the joint density *f*_***ξ***_. We identify (100 × *α*)% extreme subjects in a sample as those falling outside of $\hat{R}\left(1-\alpha \right)$. For densely observed functional data, recent studies have investigated several measures of functional outliers, such as band depth and extremal depth for functional data [53–55].

### Joint feature extraction from multiple time-varying traits

In this subsection, we describe how we perform dimension reduction for multivariate longitudinal observations by employing the covariance structure between multiple traits. Using Eq (1) and the FVE method introduced in the previous subsection, let $X^{*,[j]}\left(t\right)=\sum_{k=1}^{K^{\left[j\right]}}\xi_{k}^{\left[j\right]}\phi_{k}^{\left[j\right]}\left(t\right)$ be truncated versions of the original time-varying traits *X*^[*j*]^(*t*) using only the first *K*^[*j*]^ eigenfunctions, where $\phi_{k}^{\left[j\right]}\left(t\right)$ is the *k*-th eigenfunction of the *j*-th trait, 1 ≤ *j* ≤ *d*, 1 ≤ *k* ≤ *K*^[*j*]^, and let $\zeta_{k}^{\left[j\right]}=\xi_{k}^{\left[j\right]}/{\left(\lambda_{k}^{\left[j\right]}\right)}^{1/2}$ denote the standardized *k*-th FPC score of the *j*-th longitudinal trait, respectively. Then the functional covariance structure among the truncated time-varying traits (X*^,[*j*]^(*t*):1 ≤ *j* ≤ *d*) can be reduced to the variance-covariance matrix of $\left(\zeta_{k}^{\left[j\right]}:1\leq k\leq K^{\left[j\right]},1\leq j\leq d\right)$. Indeed, $\text{Cov}\left(X^{*,[j]}\left(s\right),X^{*,[m]}\left(t\right)\right)=\sum_{k=1}^{K^{\left[j\right]}}\sum_{\ell =1}^{K^{\left[m\right]}}{\left(\lambda_{k}^{\left[j\right]}\lambda_{\ell }^{\left[m\right]}\right)}^{1/2}\text{Cov}\left(\zeta_{k}^{\left[j\right]},\zeta_{\ell }^{\left[m\right]}\right)\phi_{k}^{\left[j\right]}\left(s\right)\phi_{\ell }^{\left[m\right]}\left(t\right)$, 1 ≤ *j* ≠ *m* ≤ *d*. This suggests to apply conventional principal component analysis (PCA) on the vector of standardized marginal FPC scores $\left(\zeta_{k}^{\left[j\right]}:1\leq k\leq K^{\left[j\right]}\right)$, 1 ≤ *j* ≤ *d*. Then, time-varying associations among multiple time-varying traits can be reproduced by a few PC scores in this second analysis. This approach has strong connections with the joint functional analysis methods of multiple random processes [56–58], and we also refer to [59] for similar ideas in a recent study on relationships between univariate and multivariate functional principal component analyses.

### Identifying subgroups for risk associated with outcomes

Our study aims to identify at-risk longitudinal growth patterns associated with undesirable outcomes. For this, we consider conditional density function of outcomes *Y* given a collection of multiple time-varying traits. Let *f*_*Y*|*S*_ be the conditional density of *Y* given a collection *S* of principal components that are obtained from the principal component analysis of the marginal FPC scores $\left(\zeta_{k}^{\left[j\right]}:1\leq k\leq K^{\left[j\right]}\right)$, 1 ≤ *j* ≤ *d*. In this study, we suggest four clusters (*S*_*m*_: 1 ≤ *m* ≤ 4) of standardized principal components of the multiple traits based on the (1 − *α*)-HDR method as follows:

$$\begin{array}{c} \begin{array}{cc} S_{1} & =\{Z\notin R\left(1-\alpha \right):|Z_{1}|/\rho_{1}^{1/2}>|Z_{2}|/\rho_{2}^{1/2},\;Z_{1}>0\},\\ S_{2} & =\{Z\notin R\left(1-\alpha \right):|Z_{1}|/\rho_{1}^{1/2}<|Z_{2}|/\rho_{2}^{1/2},\;Z_{2}>0\},\\ S_{3} & =\{Z\notin R\left(1-\alpha \right):|Z_{1}|/\rho_{1}^{1/2}>|Z_{2}|/\rho_{2}^{1/2},\;Z_{1}<0\},\\ S_{4} & =\{Z\notin R\left(1-\alpha \right):|Z_{1}|/\rho_{1}^{1/2}<|Z_{2}|/\rho_{2}^{1/2},\;Z_{2}<0\}, \end{array} \end{array}$$ (6)

where **Z** = (*Z*_1_, *Z*_2_)^⊤^ is a 2-vector consisting of the first two PC scores obtained from the PCA of ***ζ*** = (***ζ***^[1]^, ***ζ***^[2]^, ***ζ***^[3]^) and R(1-α) is the (1 − *α*)-HDR of **Z** as in Eq (4). Also, *ρ*_1_ and *ρ*_2_ are the eigenvalues associated with the first two PC scores *Z*_1_ and *Z*_2_, respectively. We then examine distributional differences among $f_{Y|S_{m}}\left(\cdot |S_{m}\right)$, 1 ≤ *m* ≤ 4, and quantify the distributional differences with analysis of variance (ANOVA).

For practical implementation, we use conditional kernel density estimators for *f*_*Y*|*S*_, given by ${\hat{f}}_{Y|S}\left(y|{\hat{S}}_{m}\right)={\left|{\hat{S}}_{m}\right|}^{-1}\sum_{i\in {\hat{S}}_{m}}K_{h}\left(Y_{i}-y\right)$ with a bandwidth *h* > 0. Here $|{\hat{S}}_{m}|$ equals the number of elements in ${\hat{S}}_{m}$, which are empirical clusters of Eq (6) defined by

$$\begin{array}{c} \begin{array}{cc} {\hat{S}}_{1} & =\{1\leq i\leq n:{\hat{Z}}_{i}\notin \hat{R}\left(1-\alpha \right),\;|{\hat{Z}}_{i1}|/{\hat{\rho }}_{1}^{1/2}>|{\hat{Z}}_{i2}|/{{\hat{\rho }}_{2}}^{1/2},\;{\hat{Z}}_{i1}>0\},\\ {\hat{S}}_{2} & =\{1\leq i\leq n:{\hat{Z}}_{i}\notin \hat{R}\left(1-\alpha \right),\;|{\hat{Z}}_{i1}|/{\hat{\rho }}_{1}^{1/2}<|{\hat{Z}}_{i2}|/{{\hat{\rho }}_{2}}^{1/2},\;{\hat{Z}}_{i2}>0\},\\ {\hat{S}}_{3} & =\{1\leq i\leq n:{\hat{Z}}_{i}\notin \hat{R}\left(1-\alpha \right),\;|{\hat{Z}}_{i1}|/{\hat{\rho }}_{1}^{1/2}>|{\hat{Z}}_{i2}|/{{\hat{\rho }}_{2}}^{1/2},\;{\hat{Z}}_{i1}<0\},\\ {\hat{S}}_{4} & =\{1\leq i\leq n:{\hat{Z}}_{i}\notin \hat{R}\left(1-\alpha \right),\;|{\hat{Z}}_{i1}|/{\hat{\rho }}_{1}^{1/2}<|{\hat{Z}}_{i2}|/{{\hat{\rho }}_{2}}^{1/2},\;{\hat{Z}}_{i2}<0\}, \end{array} \end{array}$$ (7)

where the ${\hat{Z}}_{i}$ are vectors of the first two PC scores from the PCA of ${\hat{\zeta }}_{i}=\left({\hat{\zeta }}_{i}^{\left[1\right]},{\hat{\zeta }}_{i}^{\left[2\right]},{\hat{\zeta }}_{i}^{\left[3\right]}\right)$ and $\hat{R}\left(1-\alpha \right)$ is the (1 − *α*)-HDR of ${\hat{Z}}_{i}$ as defined in the previous subsection. Also, ${\hat{\rho }}_{1}$ and ${\hat{\rho }}_{2}$ are estimates of the eigenvalues associated with the first two PC scores, respectively.

Finally, we identify subgroups for risk associated with outcomes based on multiple comparison techniques. Once we find significant differences among different subgroups, post-hoc procedures can be applied to perform multiple comparisons and control for multiple testing, which then lends support to specify risk subgroups associated with outcomes. For example, Bonferroni or Benjamini-Hochberg [60] procedures can be applied for pairwise analysis and in the next section we adopt Tukey’s post-hoc analysis [61] as a multiple comparison procedure for testing mean differences between all pairs of groups. We also use the Kruskal-Wallis rank sum test [62] as a nonparametric procedure for one-way ANOVA, and the Tukey-Kramer test (or Nemenyi test) for pairwise comparisons.

## Numerical illustrations

### Simulation study

We demonstrate the finite sample performance of the proposed method to identify clusters of extreme subjects. For this purpose random trajectories **X** = (*X*^[1]^, *X*^[2]^, *X*^[3]^) were generated such that

$$\begin{array}{c} X^{\left[j\right]}\left(t\right)=\mu_{j}\left(t\right)+\xi_{1}^{\left[j\right]}\phi_{1}^{\left[j\right]}\left(t\right)+\xi_{2}^{\left[j\right]}\phi_{2}^{\left[j\right]}\left(t\right),\;t\in \left[0,1\right], \end{array}$$ (8)

for 1 ≤ *j* ≤ 3, where the mean functions *μ*_*j*_ of *X*^[*j*]^ were zero and we use the normalized Fourier basis $\phi_{1}^{\left[j\right]}\left(t\right)=\sqrt{2}\sin\left(2\pi t\right)$ and $\phi_{2}^{\left[j\right]}\left(t\right)=\sqrt{2}\cos\left(2\pi t\right)$ on the interval [0, 1] for all 1 ≤ *j* ≤ 3. The FPC score vectors $\xi^{\left[j\right]}={\left(\xi_{1}^{\left[j\right]},\xi_{2}^{\left[j\right]}\right)}^{⊤}$ were generated with multivariate normal distributions with mean zero, sd(***ξ***^[1]^) = diag(3.0, 2.5), sd(***ξ***^[2]^) = diag(3.0, 2.0) and sd(***ξ***^[3]^) = diag(3.0, 1.5). For simplicity, we considered a common cross-covariance matrix for (***ξ***^[*j*]^, ***ξ***^[*k*]^), given by

$$\begin{array}{c} \left(\begin{array}{cc} \text{cov}(\xi_{1}^{\left[j\right]},\xi_{1}^{\left[k\right]}) & \text{cov}(\xi_{1}^{\left[j\right]},\xi_{2}^{\left[k\right]})\\ \text{cov}(\xi_{2}^{\left[j\right]},\xi_{1}^{\left[k\right]}) & \text{cov}(\xi_{2}^{\left[j\right]},\xi_{2}^{\left[k\right]}) \end{array})=(\begin{array}{cc} 0.5 & 0.1\\ 0.1 & 0.5 \end{array}\right) \end{array}$$ (9)

for 1 ≤ *j* ≠ *k* ≤ 3. Let (*ρ*_*j*_, **v**_*j*_) be (eigenvalue/eigenvector) pairs of the variance-covariance matrix Σ_***ξ***_ of ***ξ*** = (***ξ***^[1]^, ***ξ***^[2]^, ***ξ***^[3]^), satisfying *ρ*_1_ ≥ ⋯ ≥ *ρ*_6_, where det(Σ_***ξ***_) ≈ 154.8. The first two eigenvectors are **v**_1_ ≈ (0.56, 0.14, 0.57, 0.11, 0.57, 0.09)^⊤^ and **v**_2_ ≈ (−0.15, 0.75, −0.11, 0.51, −0.09, 0.38)^⊤^, and the corresponding eigenvalues are *ρ*_1_ ≈ 4.041 and *ρ*_2_ ≈ 3.068 (FVEs are 26.94% and 20.46%, respectively). Then, a scalar response *Y* was generated by *Y* = *β*_1_*Z*_1_ + *β*_2_*Z*_2_ + *ε*, where $Z_{j}=v_{j}^{⊤}\xi$, ***β*** = (*β*_1_, *β*_2_)^⊤^ = (0.4, 0.2)^⊤^ and *ε* ∼ *N*(0, 0.4^2^).

From *n* random copies (**X**_*i*_: 1 ≤ *i* ≤ *n*) of **X** for *n* = 1000, we generated sparse and noisy observations ${\tilde{X}}_{i}^{\left[j\right]}\left(T_{ijk}\right)=X_{i}^{\left[j\right]}\left(T_{ijk}\right)+\epsilon_{ijk},1\leq k\leq N_{ij}$, where the *N*_*ij*_ are randomly chosen integers between 5 and 10, *T*_*ijk*_ are iid uniform random variables on (0, 1) and *ϵ*_*ijk*_ are Gaussian measurement errors with mean zero and variance 0.1^2^, and *N*_*ij*_, *T*_*ijk*_ and *ϵ*_*ijk*_ were generated independently. Let $Y_{n}=\left\{\left(Y_{i},{\tilde{X}}_{i}^{\left[1\right]},{\tilde{X}}_{i}^{\left[2\right]},{\tilde{X}}_{i}^{\left[3\right]}\right):1\leq i\leq n\right\}$ be the random sample generated as described above, where ${\tilde{X}}_{i}^{\left[j\right]}=\left({\tilde{X}}_{i}^{\left[j\right]}\left(T_{ijk}\right):1\leq k\leq N_{i}\right)$ for 1 ≤ *j* ≤ 3.

**Fig 2:**
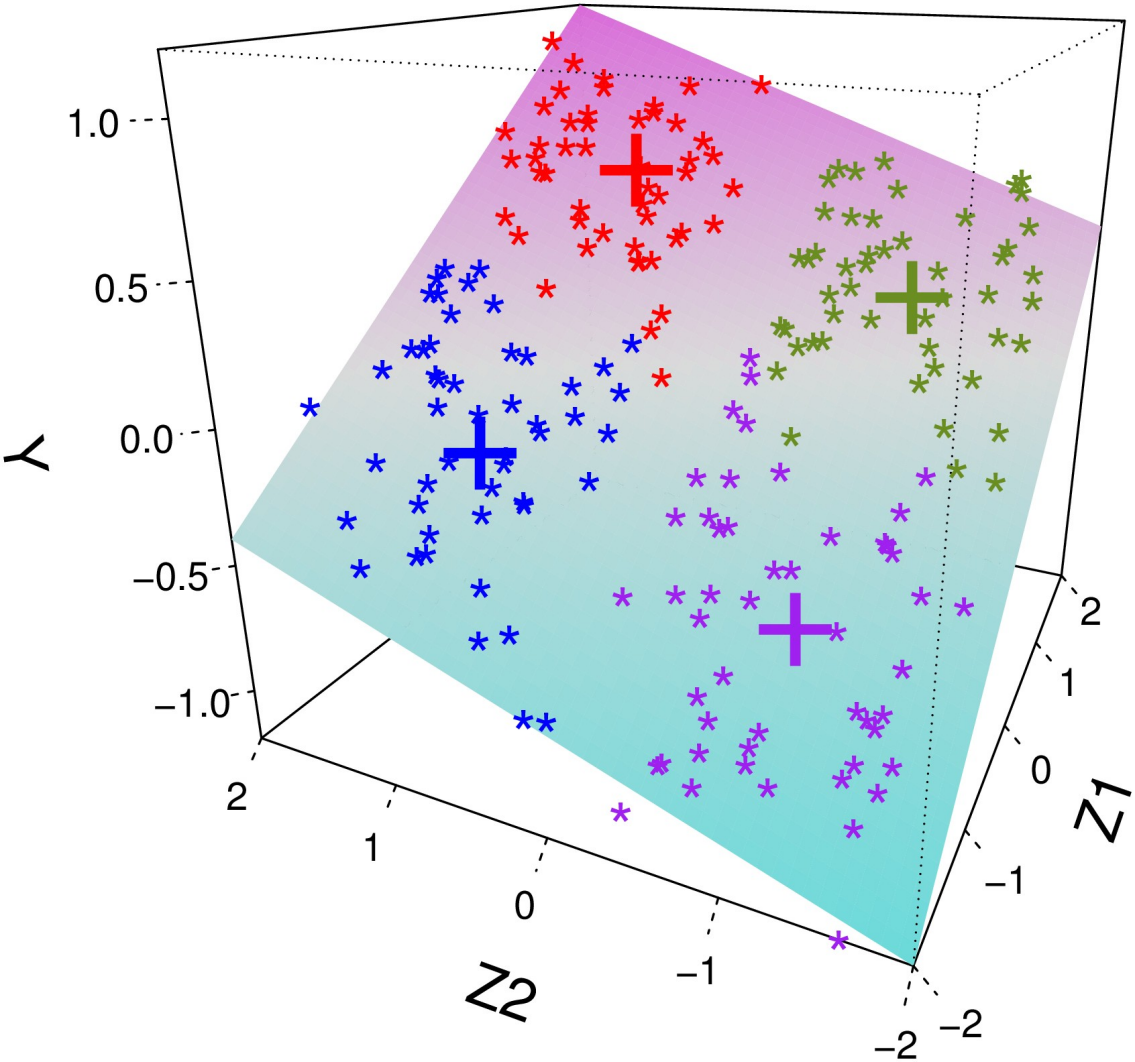
Example for visualization of observations from simulation. The proposed method identifies clusters associated with response outcomes *Y* characterized by archetypal covariate levels **Z** = (*Z*_1_, *Z*_2_), for example (high *Z*_1_, high *Z*_2_), (high *Z*_1_, low *Z*_2_), (low *Z*_1_, high *Z*_2_) and (low *Z*_1_, low *Z*_2_), which are symbolized by red, purple, green and blue points, respectively, where the crosses denote the cluster centers. The surface demonstrates the conditional mean response when regressing *Y* on **Z** = (*Z*_1_, *Z*_2_) for *n* = 200 data points.

We also demonstrate the outcomes *Y* that are associated with extremes of the predictors **Z** such that (high *Z*_1_, high *Z*_2_), (high *Z*_1_, low *Z*_2_), (low *Z*_1_, high *Z*_2_) and (low *Z*_1_, low *Z*_2_) entail different levels of response outcomes. For example, suppose that we have **Z**_1_ = (1, 1)^⊤^, **Z**_2_ = (1, −1)^⊤^, **Z**_3_ = (−1, 1)^⊤^ and **Z**_4_ = (−1, −1)^⊤^, then the corresponding conditional means of the response outcomes are 0.6, 0.2, −0.2 and −0.6 which may represent different risk levels of subgroups associated with outcomes. In Fig 2 we present an example of one i.i.d. sample from (*Y*, *Z*_1_, *Z*_2_), where we demonstrate four archetypal clusters associated with different response outcomes in different colors. This simulation setting illustrates a simple case where archetypes of functional patterns are associated with response.

Since we only have sparse and noisy longitudinal observations ${\tilde{X}}^{\left[j\right]}$ of *X*^[*j*]^, we first estimate $E(\xi^{\left[j\right]}|{\tilde{X}}^{\left[j\right]})$ for each *j*-th trajectory marginally by applying FPCA based on the conditional expectation technique (PACE) [41] to $\left({\tilde{X}}_{i}^{\left[j\right]}:1\leq i\leq n\right)$. For computation, we used the “fdapace” package in R [63], where $\left\{{\hat{\xi }}_{i}^{\left[j\right]}={\left({\hat{\xi }}_{i1}^{\left[j\right]},{\hat{\xi }}_{i2}^{\left[j\right]}\right)}^{⊤}:1\leq i\leq n\right\}$ denotes estimates of $E(\xi_{i}^{\left[j\right]}|{\tilde{X}}_{i}^{\left[j\right]})$, 1 ≤ *i* ≤ *n*, obtained from the PACE algorithm. Implementing the proposed method described in the Methodology section, we obtain four clusters ${\hat{S}}_{m}$ based on the (1 − *α*)-HDR method and standardized PC scores of multiple traits as in Eq (7). We considered the performance of our proposed methodology for the identification of risk clusters in comparison to using the univariate traits separately. Similarly, we obtained the marginal four clusters ${\hat{S}}_{m}^{\left[j\right]}$ based on the 95%-HDR method analogously to the above and standardized the individual FPC scores of each *j*-th trait as follows:

$$\begin{array}{c} \begin{array}{cc} {\hat{S}}_{1}^{\left[j\right]} & =\{1\leq i\leq n:{\hat{\xi }}_{i}^{\left[j\right]}\notin {\hat{R}}^{\left[j\right]}\left(1-\alpha \right),\;|{\hat{\xi }}_{i1}^{\left[j\right]}|/{\left({\hat{\lambda }}_{1}^{\left[j\right]}\right)}^{1/2}>|{\hat{\xi }}_{i2}^{\left[j\right]}|/{\left({\hat{\lambda }}_{2}^{\left[j\right]}\right)}^{1/2},\;{\hat{\xi }}_{i1}^{\left[j\right]}>0\},\\ {\hat{S}}_{2}^{\left[j\right]} & =\{1\leq i\leq n:{\hat{\xi }}_{i}^{\left[j\right]}\notin {\hat{R}}^{\left[j\right]}\left(1-\alpha \right),\;|{\hat{\xi }}_{i1}^{\left[j\right]}|/{\left({\hat{\lambda }}_{1}^{\left[j\right]}\right)}^{1/2}<|{\hat{\xi }}_{i2}^{\left[j\right]}|/{\left({\hat{\lambda }}_{2}^{\left[j\right]}\right)}^{1/2},\;{\hat{\xi }}_{i2}^{\left[j\right]}>0\},\\ {\hat{S}}_{3}^{\left[j\right]} & =\{1\leq i\leq n:{\hat{\xi }}_{i}^{\left[j\right]}\notin {\hat{R}}^{\left[j\right]}\left(1-\alpha \right),\;|{\hat{\xi }}_{i1}^{\left[j\right]}|/{\left({\hat{\lambda }}_{1}^{\left[j\right]}\right)}^{1/2}>|{\hat{\xi }}_{i2}^{\left[j\right]}|/{\left({\hat{\lambda }}_{2}^{\left[j\right]}\right)}^{1/2},\;{\hat{\xi }}_{i1}^{\left[j\right]}<0\},\\ {\hat{S}}_{4}^{\left[j\right]} & =\{1\leq i\leq n:{\hat{\xi }}_{i}^{\left[j\right]}\notin {\hat{R}}^{\left[j\right]}\left(1-\alpha \right),\;|{\hat{\xi }}_{i1}^{\left[j\right]}|/{\left({\hat{\lambda }}_{1}^{\left[j\right]}\right)}^{1/2}<|{\hat{\xi }}_{i2}^{\left[j\right]}|/{\left({\hat{\lambda }}_{2}^{\left[j\right]}\right)}^{1/2},\;{\hat{\xi }}_{i2}^{\left[j\right]}<0\}, \end{array} \end{array}$$ (10)

where ${\hat{R}}^{\left[j\right]}\left(1-\alpha \right)$ is the (1 − *α*)-HDR of ${\hat{\xi }}_{i}^{\left[j\right]}$.

We report the simulation results in Table 1, where the numbers of joint extreme trajectory clusters associated with outcomes obtained from 1000 Monte Carlo repetitions with sample size *n* = 1000 are shown for *α* = 0.05. Tukey’s post-hoc multiple comparison was employed to determine how many associated clusters exist at each Monte Carlo run. That is, at each repetition, we counted the subgroups which are completely separated by Tukey’s post-hoc analysis. By comparing conditional mean differences of outcomes between the four extreme clusters, we found that the proposed method identified more risk clusters than the marginal methods which detected two clusters on average for all cases. The joint method identified the three or four of the archetype clusters which depict (high *Z*_1_, high *Z*_2_), (high *Z*_1_, low *Z*_2_), (low *Z*_1_, high *Z*_2_) and (low *Z*_1_, low *Z*_2_) up to 90.4% (= 59.8% + 30.6%). This result supports the use of multiple trajectories instead of a single trajectory when identifying archetypes of risk sets. This applies even as the first two PC scores have less than 50% FVE, as in this simulation example.

**Table 1:** Simulation results for identification of at-risk multiple trajectories clusters associated with response outcomes.

Number of risk clusters associated with outcomes	Marginal method			Joint method PC-FPC
	*j* = 1	*j* = 2	*j* = 3	
< 2	3.6%	5.0%	4.7%	0.0%
2	62.3%	83.0%	84.0%	9.6%
3	31.7%	11.5%	10.8%	59.8%
4	2.4%	0.5%	0.5%	30.6%

For 1000 Monte Carlo (MC) repetitions with sample size *n* = 1000, the numbers of risk clusters were identified by analysis of variance (ANOVA) and Tukey’s multiple comparisons with a family-wise significance level 0.05. At each repetition, we counted subgroups completely separated by Tukey’s post-hoc analysis. For example, we identify two clusters if all subgroups included in a cluster show significant differences in pairwise comparison (family-wise significance level 0.05) against the other cluster members. Percentages in each column of the table demonstrate how many clusters are detected through 1000 MC repetitions. For the comparison with the marginal method, we applied the same procedure, using the marginal trajectory information only for *j* = 1, 2, 3, respectively.

### Analysis of PROBIT data

#### Marginal analysis for longitudinal measurements of growth traits

**Fig 3:**
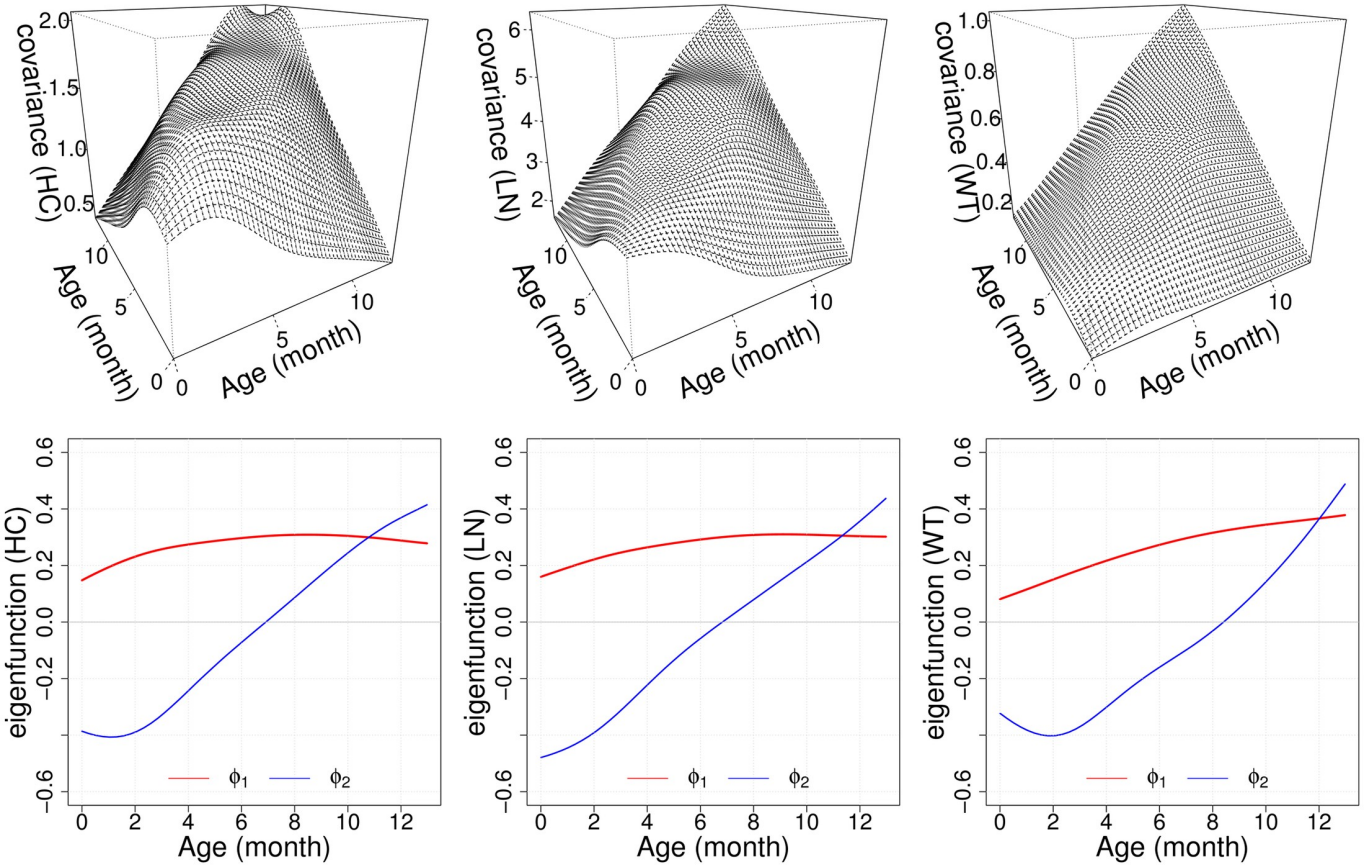
Estimated auto-covariance functions and eigenfunctions. Estimated auto-covariance functions *G*(*s*, *t*) (top) and the corresponding first two eigenfunctions *ϕ*_1_(*t*) and *ϕ*_2_(*t*) (bottom) as in Eq (3) for head circumference (HC, left), body length (LN, middle) and weight (WT, right), respectively. Eigenfunctions represent the qualitative factors “General growth” (red) and “Growth acceleration” (blue). The cumulative fractions of variation explained (FVE) of the first two components are 97.70%, 96.92% and 98.14% for HC, LN and WT, respectively.

PROBIT contains three main time-varying traits; head circumference (HC), body length (LN) and weight (WT). For the marginal FPCA of these three variables, we applied the PACE technique introduced in the Methodology section, since we only have sparse and irregular observations available. As in the simulation study, we also used the “fdapace” package in R [63]. Auto-covariance functions of each time-varying trait were reconstructed by the first two eigenfunctions. The fractions of variance explained (FVEs) were 97.70%, 96.92% and 98.14% for HC, LN and WT, respectively. See Fig 3 for illustrations of estimated auto-covariance functions and eigenfunctions. The first and second eigenfunctions can be regarded as “General growth” and “Growth acceleration”, respectively. Based on the observed high FVE coverages, we assume in the rest of the paper that these two qualitative features carry information about the longitudinal patterns of time-varying growth traits in the PROBIT data.

**Fig 4:**
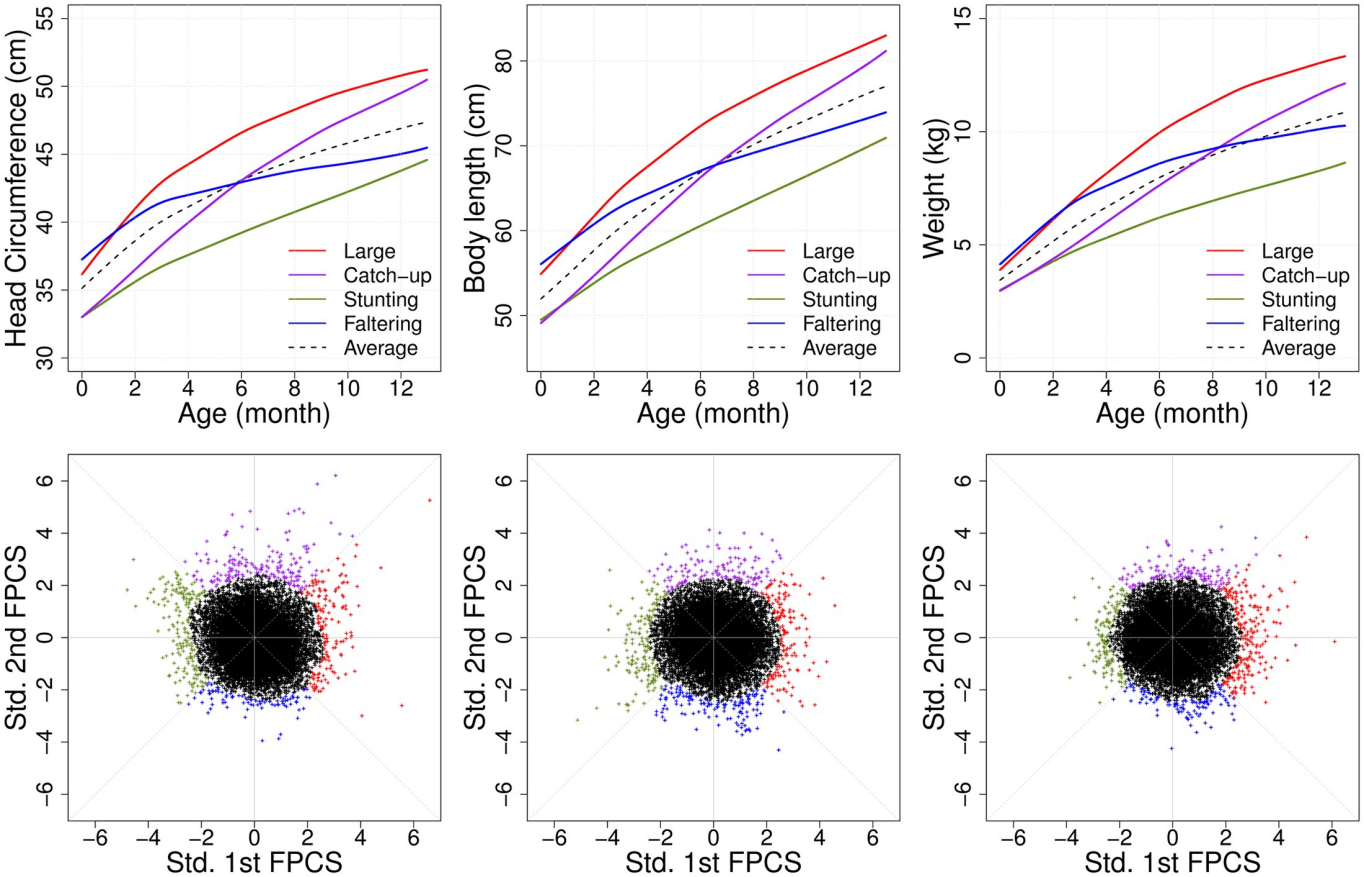
Extreme functional patterns from marginal analysis. Head circumference (HC, left), body length (LN, middle) and weight (WT, right) traits, respectively. Four outlying clusters (falling into the smallest 5% of the bivariate density) are demonstrated with respective different colors, with estimated mean curves corresponding to the four outlying subgroups, respectively, which represent the four qualitative longitudinal growth patterns: “Generally Large” (red), “Catch-up” (purple), “Stunting” (green) and “Faltering” (blue) (top panels). The corresponding scatterplots for the first two functional principal component scores (bottom).

The marginal analysis of outlying subgroups was performed with a (1 − *α*)-high density region (HDR) with *α* = 0.05. Subjects were classified as “normal” if they belonged to the 95% support region in the HDR criterion as in Eq (4), while outlying subjects were classified as 5% extreme cases falling outside the HDR criterion. In this study, we considered four exclusive subgroups as in Eq (10), where the trait index [*j*], 1 ≤ *j* ≤ 3, stands for HC, LN and WT, respectively. The four outlying subgroups correspond to distinctive growth patterns, which can be labeled as “Generally Large”, “Catch-up”, “Stunting” and “Faltering” (Fig 4). We found that the outlying patterns were discordant across traits. For example, subjects who were classified into the generally large head circumference subgroup could be normal for body length or weight, and vice versa. Moreover, as there are 4^3^-combinations of subgroups entailed by the marginal analysis, it is difficult to associate all these multiple trajectory patterns with the response of interest, which is IQ at 6.5 years.

#### Potential risk subgroups for cognitive development

We constructed joint outlying subgroups of multiple time-varying traits based on the HDR method and standardized PC scores of multiple traits $\left({\hat{\zeta }}_{i}^{\left[j\right]}={\hat{\xi }}_{ik}^{\left[j\right]}/{\left({\hat{\lambda }}_{k}^{\left[j\right]}\right)}^{1/2}:1\leq k\leq 2,1\leq j\leq 3\right)$ as described in Eq (7). Principal component analysis results for the six FPC scores are presented in Table 2, where the first and second FPC stand for scores of general growth and growth acceleration, respectively. We found that these two features were captured in the first two PC loadings. In this study, we focus on the first two PC scores as they explain more than 95% of the variation for each of the three modalities [41, 42, 45].

**Table 2:** Principal component analysis of functional principal components.

qualitative feature of joint FPCs	marginal FPC factor	PC loadings					
		PC1	PC2	PC3	PC4	PC5	PC6
General growth	HC-FPC1	**0.502**	0.155	0.457	0.690	0.044	0.191
	LN-FPC1	**0.552**	0.198	-0.289	-0.430	0.243	0.574
	WT-FPC1	**0.527**	0.331	-0.109	-0.182	-0.383	-0.649
Growth acceleration	HC-FPC2	-0.216	**0.517**	0.688	-0.422	0.188	-0.020
	LN-FPC2	-0.185	**0.546**	-0.432	0.324	0.570	-0.227
	WT-FPC2	-0.291	**0.512**	-0.190	0.154	-0.657	0.402
partial FVE		0.382	0.261	0.122	0.094	0.088	0.053
cumulative FVE		0.382	0.643	0.765	0.859	0.947	1.000

Principal component analysis for the variance-covariance matrix of the first two marginal functional principal component scores (FPCs) for head circumference (HC), body length (LN) and weight (WT).

On the other hand, socio-economic factors affect childhood intelligence in ways that are not reflected in the FPCA of time-varying traits [26, 27]. To avoid confounding effects by socio-economic variables, we used a linear mixed effects model to reduce the influence of the socio-economic indicators. Hospital information was treated as a random effect as it is related to the random clustered design of the PROBIT study. We used the residuals of the linear mixed effects model and marginalized the effect of the potentially confounding variables considered above. For details of the data preprocessing, we refer to [22] and [13].

**Fig 5:**
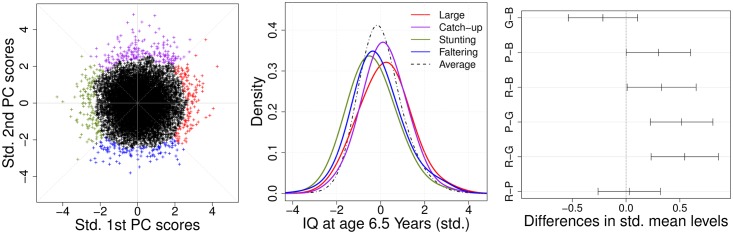
Extreme subgroup identification from the proposed method. (Left panel) Scatterplot of 95% high density region clustering from principal component analysis for head circumference, body length and weight and (Middle panel) the corresponding conditional kernel density estimates for long-term intelligence for each subgroup. Here, qualitative growth patterns include “Generally Large” (red), “Catch-up” (purple), “Stunting” (green) and “Faltering” (blue), whereas the black dashed line represents the “normal” subgroup that consists of subjects who do not belong to the four outlying subgroups. (Right panel) Tukey’s multiple comparisons of mean differences for standardized IQ along outlying subgroups are demonstrated by family-wise 95% confidence intervals, where we label the four subgroups as R (red, “Generally Large”), P (purple, “Catch-up”), G (green, “Stunting”) and B (blue, “Faltering”).

In Fig 5, we find that conditional densities of IQ measured at 6.5 years, given the joint outlying subgroups, exhibited different distributional behaviors. The four subgroups were constructed by a principal component analysis of the standardized six FPC scores for HC, LN and WT. The significance of the group mean differences was examined by one-way ANOVA (p-value = 0.002), and also by the Kruskal-Wallis rank sum test [62], a nonparametric procedure for one-way ANOVA (p-value < 0.001), so that the results were qualitatively the same. For a more detailed comparison, we performed post-hoc analysis with Tukey’s multiple comparison procedure. As shown in Table 3 and Fig 5, we found significant mean differences among outlying subgroups in a family-wise 5%-level test. “Stunting” and “Faltering” were associated with higher risk in comparison with the “Generally Large” and “Catch-up” subgroups. Similar results were obtained by using the nonparametric procedure for pairwise comparisons of the Tukey-Kramer test. We also applied several multiple comparison techniques such as Bonferroni and Benjamini-Hochberg methods for post-hoc analysis and similar results were obtained by controlling false discovery rate (FDR) at 5%. For example, we found that “Stunting” and “Faltering” were associated with higher risk in comparison with the “Generally Large” and “Catch-up” subgroups after application of both procedures. The results were suggestive of higher risk for “Faltering” versus “Catch-up” (p-value = 0.060 after Bonferroni correction).

**Table 3:** One-way ANOVA for subgroup detection.

variation	df	sum of sq.	mean sq.
subgroup	3	27.002	9.001
residuals	598	631.902	1.057
total	601	658.904	
F-value = 8.518		p-value < 0.001	

One-way ANOVA result provides evidence for differences in group means of long-term IQ for the four outlying subgroups “Generally Large”, “Catch-up”, “Stunting” and “Faltering”.

**Fig 6:**
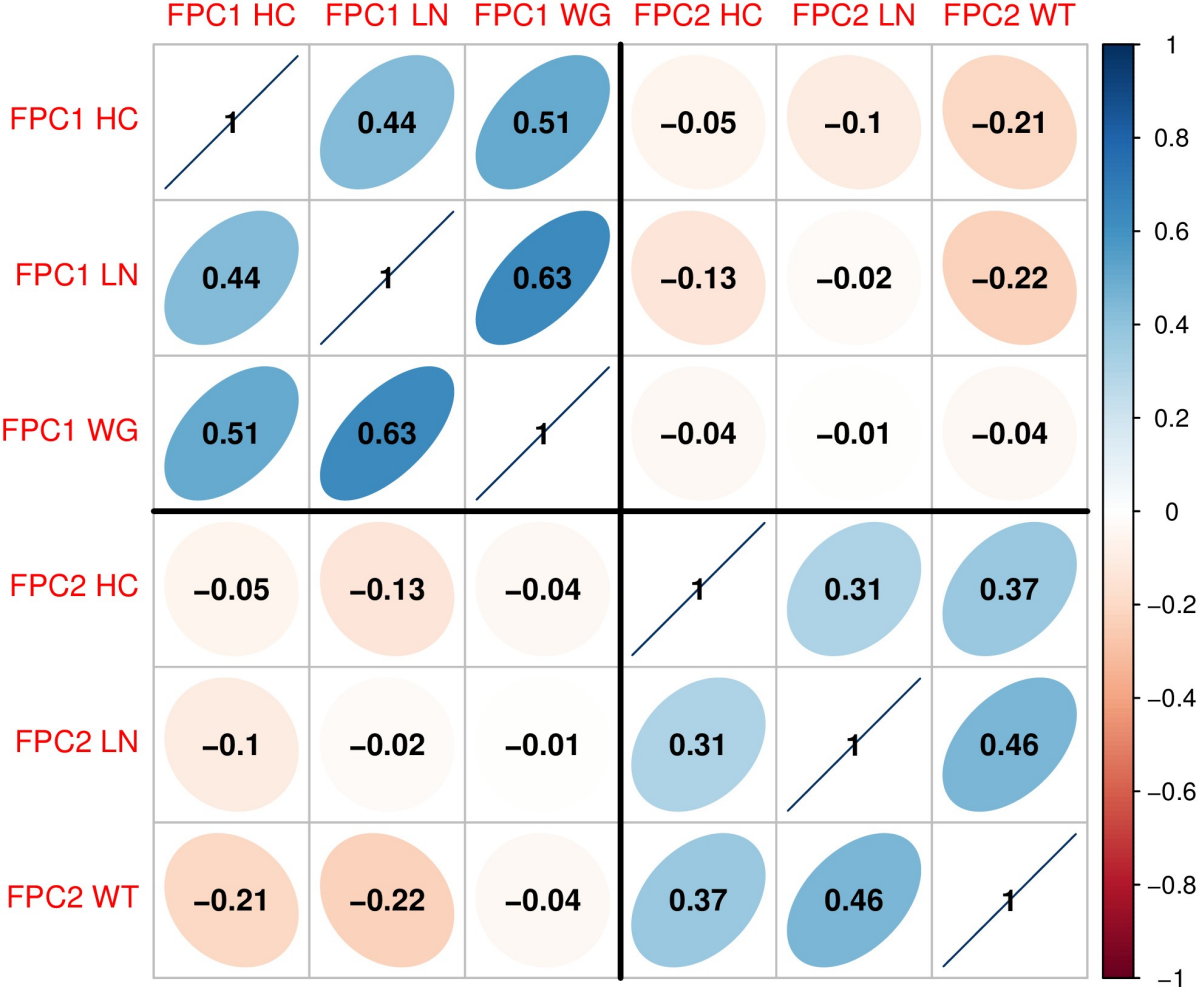
Correlation plot. Correlations between the first two functional principal component (FPC) scores of head circumference (HC), body length (LN) and weight (WT). FPC scores among the first and second components have positive correlations, respectively, which suggests to combine the three growth features linearly with PC loadings as in Table 2.

**Fig 7:**
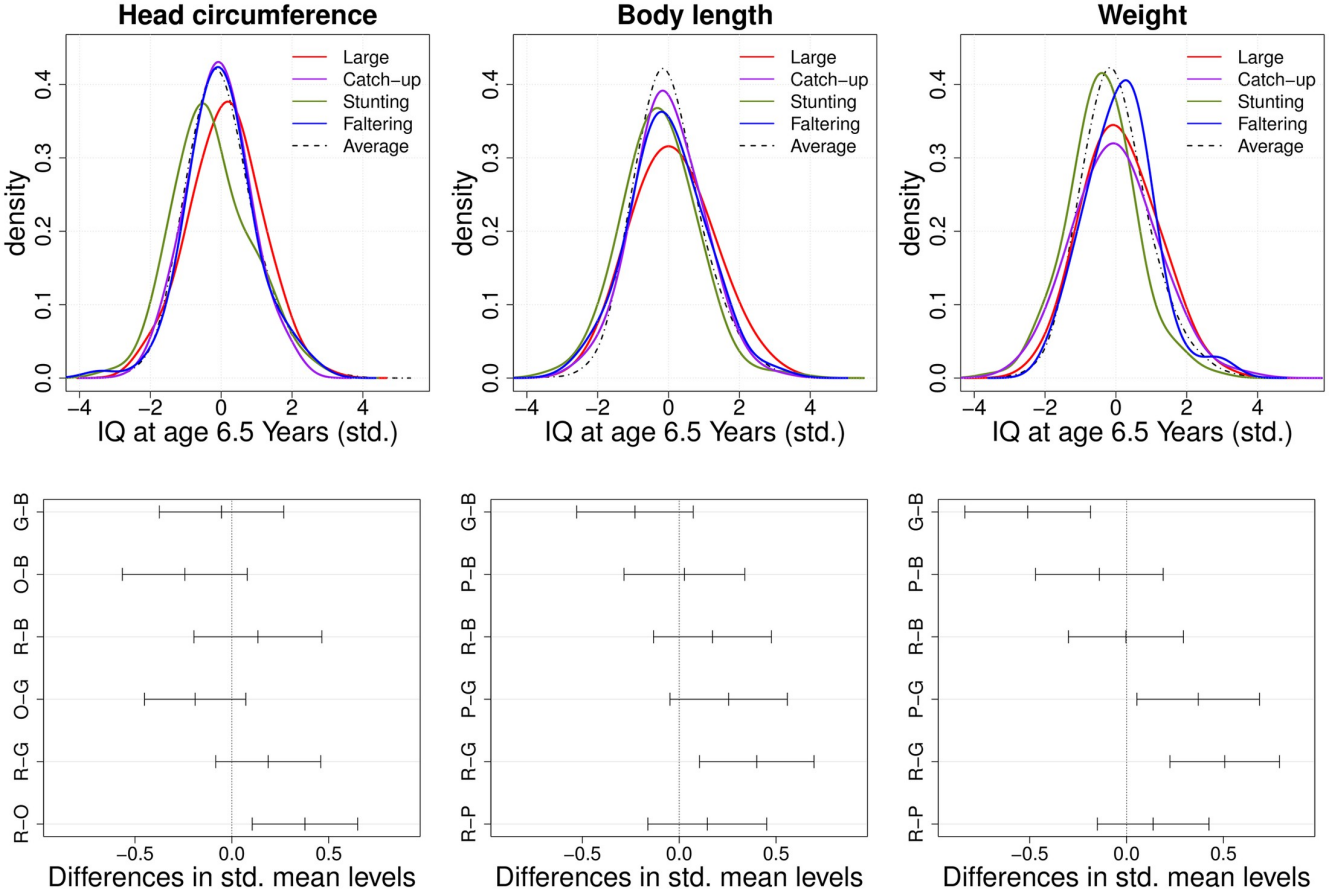
Marginal subgroup identification. (Top panels) Conditional kernel density estimators and (bottom panels) illustrations of Tukey’s multiple comparisons for standardized IQ along outlying subgroups. As in Fig 5, the first two functional principal component (FPC) scores are used to construct subgroups for head circumference (left), body length (middle) and weight (right), labeled R (red, “Generally Large”), P (purple, “Catch-up”), G (green, “Stunting”) and B (blue, “Faltering”).

We close this section with a short remark on Figs 6 and 7 which presents the result of the marginal analysis as described in the Simulation study section. In contrast to the proposed joint method, we found that the marginal procedures may not effectively detect risk growth patterns associated with long-term IQ outcomes. From the post-hoc analysis, “Generally Large” and “Stunting” subgroups had significantly different IQ performance for head circumference (p-value = 0.002), body length (p-value = 0.003) and weight (p-value < 0.001), but no significant difference was found between other subgroups such as “Catch-up” or “Faltering” for head circumference and body length. We note that “Stunting” for one of the marginal components may not be an at-risk growth pattern associated with IQ development compared to the other subgroups. Moreover, “Faltering” for weight showed higher IQ performances than the “Stunting” subgroup (p-values < 0.001), while failure to thrive in infancy, defined as weight faltering in the first 9 months of life, was previously found to be associated with persistent deficits in intellectual development when measured at 8 years [64]. These results suggest that the combination of multiple growth patterns can indeed be beneficial for identifying risk subgroups associated with IQ outcomes.

## Discussion

This paper outlines a statistical framework for exploring multivariate functional patterns deduced from sparsely and irregularly sampled longitudinal data and their association with long-term outcomes. For the joint analysis of children’s growth and IQ in the PROBIT data, we propose a straightforward way to combine multiple growth indicators under a single framework. We extract multiple growth features jointly, by using standard multivariate analysis of the functional principal components. The major modes of growth variation are then represented at the subject level and we can thus profile outlying multiple growth patterns, which can be considered as archetypes of growth.

The focus of this paper is how to combine multivariate functional data to identify extremal curve patterns and associate these features with responses. One may consider an alternative application of multivariate functional principal component analysis as in [58, 59, 65, 66] or functional ANOVA [67–69] as alternatives. However, for all approaches it is critical how to determine outlying and extremal patterns jointly from multiple functional data, and the high-density region (HDR) method to detect outlying functional principal components that we adopt here is a natural extension of similar nonparametric approaches in multivariate data analysis. Recently several related studies have been introduced for functional outlier detection [54, 70, 71] but it still remains an open problem how to combine these with other methodologies such as clustering and archetypal analysis.

In the PROBIT growth data analysis, we identified four archetypal subgroups of infant growth patterns, namely “Stunting”, “Faltering”, “Generally Large” and “Catch-up”. In addition we also found that covariance structures have marginally similar patterns across the functional traits considered; head-circumference, body length and weight. According to our analysis, subgroups corresponding to “Stunting” and “Faltering” in the infant period had lower downstream IQ compared to “Generally Large” and “Catch-up” subgroups. This finding is supported by previous studies that link deficient infant growth and later life cognitive performance degradation.

It is worth mentioning that single growth indicators were not found to be associated with risk of lowered IQ, and the marginal analysis of single growth traits did not produce informative results in the PROBIT analysis (See Fig 7). Also, there is a possibility that the absence of any measure of cognitive ability during infancy in the data could be explained by reverse causality, namely, poor cognitive function in infancy may have led to worse dietary intake. Both may have been consequences of poor parenting or unmeasured insults during pregnancy or infancy. The proposed methods are not limited to specific data structures, such as growth data, but can be applied to many other kinds of longitudinal data as well, whenever a downstream outcome is of interest.
